# State Medical Association of Texas

**Published:** 1908-04

**Authors:** W. B. Russ, Holman Taylor

**Affiliations:** State Medical Association of Texas; Chairman, San Antonio, Texas; Secretary, Marshall, Texas


### State Medical Association of Texas.

FEBRUARY 1, 1908.

DEAR DOCTOR: The enclosed pamphlet [see Dr. Foscue's letter, ED.], giving to the various classes of applicants explicit directions how to comply with the new Medical Practice Act, is respectfully submitted to you for your consideration.

The year of grace, allowed by this act for the procuring of verification licenses, will expire July 12, 1908. All who have not by that date procured and registered verification licenses will be forced either to stop practicing medicine or go before the Board for examination.

The Board of Councilors has assured itself that this law is entirely constitutional and will be rigidly enforced. For any worthy practitioner of medicine, either from wilful neglect, carelessness or ignorance of requirements, to fail to qualify under the law would be exceedingly unfortunate. The Board takes this means to inform and aid the profession, at the same time desiring to preclude unnecessary and unfortunate litigation. The law benefits the public and the doctor as well, as it will debar many who are now practicing on certificates fraudulently obtained.

If you have not already done so, you are urged to *immediately* take such steps as are necessary to secure your verification license, and, when secured, to have the same *recorded* in the office of the district clerk of your county. For any additional information, address Dr. G. B. Foscue, Waco, Secretary of the Examining Board.

Fraternally yours,

BOARD OF COUNCILORS,
STATE MEDICAL ASSOCIATION OF TEXAS.

W. B. RUSS, M. D., Chairman, San Antonio, Texas.
HOLMAN TAYLOR, M. D., Secretary, Marshall, Texas.

